# Placing Abstract Concepts in Space: Quantity, Time and Emotional Valence

**DOI:** 10.3389/fpsyg.2018.02169

**Published:** 2018-11-14

**Authors:** Greg Woodin, Bodo Winter

**Affiliations:** Department of English Language and Applied Linguistics, University of Birmingham, Birmingham, United Kingdom

**Keywords:** spatial cognition, metaphor, mental representation, abstract thought, numerical cognition

## Abstract

Research has shown that abstract concepts are often conceptualized along horizontal and vertical axes. However, there are mixed results concerning which axis is preferred for which type of conceptual domain. For instance, it has been suggested that the vertical axis may be preferred for quantity in tasks using linguistic stimuli (e.g., ‘more,’ ‘less’), whereas numerals (e.g., ‘1,’ ‘2,’ ‘3’) may be more prone to horizontal conceptualization. In this study, we used a task with free response options to see where participants would place quantity words (‘most,’ ‘more,’ ‘less,’ ‘least’), numerals (‘2,’ ‘4,’ ‘7,’ ‘9’), time words (‘past,’ ‘future,’ ‘earliest,’ ‘earlier,’ ‘later,’ ‘latest’) and emotional valence words (‘best,’ ‘better,’ ‘worse,’ ‘worst’). We find that for quantity words, the vertical axis was preferred; whereas for numerals, participants preferred the horizontal axis. For time concepts, participants preferred the horizontal axis; and for emotional valence, they preferred the vertical axis. Across all tasks, participants tended to use specific axes (horizontal, vertical), rather than combining these two axes in diagonal responses. These results shed light on the spatial nature of abstract thought.

## Introduction

Space is a powerful resource which humans habitually make use of to understand abstract concepts such as time. Proponents of Conceptual Metaphor Theory (e.g., Lakoff and Johnson, 1980; Gibbs, 1994; Kövecses, 2002) and embodied cognition more generally (e.g., Barsalou, 1999; Wilson, 2002; Glenberg et al., 2014) have long emphasized that abstract concepts may be grounded in terms of concrete ones, such as space.

Our reliance on space for abstract thought is reflected in the language we use to talk about quantity, time and emotional valence; for example, English speakers speak of ‘high’ and ‘low’ numbers, look ‘forward’ to future events and look ‘back’ on past ones, and profess to feel either ‘up’ or ‘down’ (Lakoff and Johnson, 1980; Casasanto and Boroditsky, 2008; Winter et al., 2015a). Moreover, in our everyday lives we are surrounded by spatial graphical representations of abstract concepts, such as data visualizations, number lines and timelines (Tversky, 2011). The use of horizontal and vertical axes in particular has been shown to be prolific in the grounding of abstract concepts, leading us to imagine time flowing from left to right, for instance, or to conceptualize quantities as increasing upward through space (e.g., Tversky et al., 1991; Ishihara et al., 2008; Hartmann et al., 2012). In this paper, we focus on spatial conceptualizations of quantity, time, and emotional valence. Each of these conceptual domains has been investigated individually, but very few studies have studied spatial conceptualizations across these domains (for an exception, see Tversky et al., 1991).

Research on the mental representation of quantity suggests that people conceptualize quantities along the horizontal axis on a ‘mental number line,’ where smaller numbers are positioned to the left and larger numbers to the right. A seminal finding in this field is the so-called Spatial-Numerical Association of Response Codes (SNARC) effect, where relatively smaller numbers are responded to more quickly with the left hand, and relatively larger numbers are responded to more quickly with the right hand (Dehaene et al., 1993; Wood et al., 2008; Chinello et al., 2012; Fischer and Shaki, 2014). Similar horizontal effects have been found with eye movements, where the sequential processing of a relatively large number followed by a relatively small number triggers leftward eye movements (Loetscher et al., 2008). In addition, when people are instructed to generate a sequence of numbers randomly, leftward eye movements predict that the next number they will generate will be smaller (Loetscher et al., 2010).

Evidence has also accumulated for vertical conceptualizations of quantity. In accordance with visual representations such as bar charts (where higher vertical positions correspond to ‘more’), and English expressions such as ‘plummeting shares’ and ‘soaring costs’ (Lakoff and Johnson, 1980), researchers have found that SNARC-like effects can also be obtained with vertical response setups, where participants respond more quickly to relatively larger numbers using a vertically higher response button (Hartmann et al., 2014). Furthermore. Hartmann et al. (2012) found that when participants were asked to randomly generate numbers, they generated comparatively larger numbers when being moved vertically upward than when being moved downward. Similarly, Winter and Matlock (2013) found that randomly generated numbers were larger when participants looked upward as opposed to downward (for a review of the literature on horizontal and vertical quantity effects, see Winter et al., 2015b).

Just as is the case for quantity, researchers have found that time is conceptualized along both horizontal and vertical dimensions. For example, Ishihara et al. (2008) found that when participants were asked to indicate whether the timing of an auditory stimulus was earlier or later than a preceding stimuli, they were faster to respond to earlier stimuli with a left-side button, and to later stimuli with a right-side button. In analogy to SNARC, this effect has come to be known as STEARC, the Spatial-Temporal Association of Response Codes (see also Vallesi et al., 2008, 2011). Similar left-right associations have been reported with short and long stimuli durations (Conson et al., 2008), and past- and future-related concepts, such as days of the week (Gevers et al., 2003, 2004) (for a review, see Bonato et al., 2012). More limited evidence suggests the existence of vertical representations of time. For instance, Ruiz Fernandéz et al. (2014) found that participants were quicker to respond to a square positioned in upper space when it was paired with a future-related word, and to a square positioned in lower space when it was paired with a past-related word. Furthermore, Leone et al. (2018) found that many participants ordered time concepts (past, present and future) chronologically upward, although most participants still preferred a left-to-right representation.

Finally, research suggests that emotional valence (good versus bad) may also be represented spatially. For instance, English speakers use expressions such as ‘cheer up’ and ‘down in the dumps’ (Lakoff and Johnson, 1980), and evidence suggests that cognition may reflect this vertical association, with Meier and Robinson (2004) reporting that participants responded more quickly to positive words such as ‘pride’ when these words were presented in a higher position on a computer screen. More recent studies have revealed similar effects, showing that upward vection biases the recall of more positive memories, whereas downward vection has the opposite effect (Casasanto and Dijkstra, 2010; Seno et al., 2013). In terms of horizontal space, Casasanto’s (2009) body-specificity hypothesis proposes that right-handers will associate the more dominant right side of their bodies (and therefore right-side space) with more positive emotions, whereas left-handers will exhibit the reverse association. In support of this hypothesis, Casasanto found that right-handers placed ‘good’ items into a right-positioned square and ‘bad’ items into a left-positioned square, while left-handers did the opposite (see also Casasanto and Jasmin, 2010; Casasanto and Chrysikou, 2011; Casasanto and Henetz, 2012).

If quantity, time and emotional valence can be conceptualized both horizontally and vertically, which axis will be preferred when both axes are available? The majority of studies reported above (e.g., Dehaene et al., 1993; Meier and Robinson, 2004; Ishihara et al., 2008) were not equipped to deal with this question because they restricted response options to a single axis (see Fischer and Campens, 2009; Walker and Cooperrider, 2016). However, one classic study that did permit participants free choice of response was conducted by Tversky et al. (1991). In this study, participants were asked to place stickers that they were told represented temporally ordered events (‘breakfast,’ ‘lunchtime,’ ‘dinner’) onto a page. Participants were also asked to place stickers representing quantity-related concepts (amounts of sand, body height measurements) and emotional valence-related concepts (liked and disliked foods and television shows). Results indicated that English participants were more likely to structure time responses horizontally from left to right, and quantity and emotional valence responses vertically from down to up.

The dominance of the horizontal axis for time has since been corroborated by other studies (e.g., Boroditsky, 2001; Fuhrman et al., 2011; Leone et al., 2018), and those investigating emotional valence have confirmed stronger vertical than horizontal effects (e.g., Crawford et al., 2006; Brunyé et al., 2012; Damjanovic and Santiago, 2016). With our study, we aimed to replicate a dominant horizontal effect for time and a dominant vertical effect for emotional valence in a novel task allowing comparison across all three domains (quantity, time, emotional valence).

Meanwhile, research into quantity has reported conflicting results. For example, some studies report the vertical axis to be dominant (Winter and Matlock, 2013), and others the horizontal (Fischer and Campens, 2009; Holmes and Lourenco, 2012). One explanation for this lack of consensus is suggested by Winter et al. (2015b), who discuss circumstantial evidence indicating that vertical conceptualizations of quantity may be preferred for linguistic stimuli. For instance, vertical as opposed to horizontal effects were obtained by Sell and Kaschak (2012) in a task where participants read sentences containing quantity information (e.g., ‘More/less runs were being scored this game’). Moreover, other studies that found reliable vertical effects also used linguistic stimuli (Guan et al., 2013; Lachmair et al., 2014). An alternative explanation is provided by Pecher and Boot (2011), who suggest that the vertical axis should be preferred for quantities presented in physical, real-life contexts (e.g., ‘seven pairs of shoes’), rather than numerals presented in isolation (e.g., ‘7’). In the current study, we assessed which axis is dominant in the domain of quantity for both linguistic stimuli (e.g., ‘most,’ ‘least’) and exact numerals (e.g., ‘2,’ ‘9’). Our use of individual quantity words allowed us to test whether vertical conceptualisations of quantity persist when these words are divorced from physical context.

Finally, we look more specifically at the possibility of diagonal representations. Research allowing participants free choice of response (e.g., Tversky et al., 1991; Fischer and Campens, 2009; Leone et al., 2018) opens the door to representations where the traditional axes are eschewed in favor of non-linear responses, or where both horizontal and vertical axes are utilized at the same time (diagonal responses). This latter possibility is supported by Walker and Cooperrider’s (2016) ‘continuity of metaphor’ hypothesis, which suggests that different axial representations may be compatible with each other and so may be co-activated. In support of this hypothesis, the authors report that when speakers gesture about time concepts, they often move their hands both forward (sagittal space) and to the right (horizontal space) when discussing the future, and backward and to the left when discussing the past. Our paper examines diagonal responses in light of this ‘co-activation’ hypothesis.

Our study used a variation of the methodology used by Tversky et al. (1991) to investigate conceptualisations of quantity, time and emotional valence using free response options. In all of our experiments, we asked participants to position words pertaining to quantity, time and emotional valence in a two-dimensional plane. Chiefly, we wanted to see which axis (horizontal or vertical) would be preferred if both were available. Previous research (e.g., Tversky et al., 1991; Holmes and Lourenco, 2012) led us to predict that the horizontal axis with a left-to-right orientation would be preferred for time and numerals, whereas the vertical axis with a down-to-up orientation would be preferred for emotional valence, as well as vague quantities presented linguistically in the form of quantity words (e.g., ‘more,’ ‘less’). Our setup also allowed us to test Walker and Cooperrider’s (2016) hypothesis that participants might conceptualize each domain using both horizontal and vertical axes simultaneously, which we interpreted as being represented by a diagonal response. Finally, we incorporated a qualitative interview component into our tasks to investigate our participants’ motivations behind their responses. We hoped that these interview responses would help enlighten the factors underlying horizontal and vertical associations of quantity, time and emotional valence.

Experiments 1 and 2 test the conceptualization of quantities presented linguistically, whereas Experiment 3 contrasts these results with a task involving numerals. Across Experiments 1 and 2, we also assess how participants place time- and emotional valence-related words. Experiment 4 provides a replication-extension for all four domains (quantity, numerals, time and emotional valence) using a computerized task.

## Experiment 1

Fifty native English-speaking adults (27 male, 23 female; 42 right-handed, 8 left-handed) volunteered to participate in the study.

### Procedure

Participants completed three tasks (quantity, time, emotional valence). In each task, participants were presented with a piece of A4 paper positioned flat on a table. The center of the page contained a response box with a pair of centrally positioned axes, resembling a square containing a plus sign. Written instructions were presented above this box. The inclusion of axes in our response area diverged from Tversky et al. (1991), who used blank paper.

We asked participants to first place words ‘best’ and ‘worst’ (emotional valence), then ‘most’ and ‘least’ (quantity), and then ‘past’ and ‘future’ (time) (the order of tasks was fixed). Participants marked each word with a pen using the initial of each word (e.g., ‘L’ for ‘least’). The word order used in these instructions was counter-balanced across participants. For instance, in the time task, half the participants (*N* = 25) received the instruction ‘Mark P for PAST and F for FUTURE,’ whereas half received ‘Mark F for FUTURE and P for PAST.’

Following the main task, a semi-structured interview was conducted and recorded using an Android smartphone app. Participants were encouraged to explain their responses to each task. They were also invited to elaborate on any interesting themes their responses revealed.

### Statistical Analysis

All data discussed hereafter was analyzed within the R statistical programming environment, version 3.3.1 (R Core Team, 2016). The packages ‘tidyverse’ version 1.1.1 (Wickham, 2017b) and ‘stringr’ 1.2.0 (Wickham, 2017a) were used for data processing. The packages ‘lme4’ version 1.1.15 (Bates et al., 2015) and ‘afex’ 0.19.1 (Singmann et al., 2018) were used for mixed models. Finally, the package ‘lsr’ 0.5.0 (Navarro, 2015) was used to compute Cramér’s *V*.

All analysis and code are made available via the following Open Science Framework repository: https://osf.io/48u5q/.

### Results

#### Quantitative Results (Placement Task)

For ease of discussion, we will refer to ‘most,’ ‘future,’ and ‘best’ responses as ‘positive’ labels, whereas ‘least,’ ‘past,’ and ‘worst’ we will refer to as ‘negative’ labels (not to be confused with good or bad with respect to emotional valence). Paper responses were coded for what we will call the ‘Dominant Orientation’ chosen by the participant (i.e., whether the labels were aligned horizontally, vertically or diagonally). In addition, we coded for what we will call the ‘Dominant Direction’ (i.e., whether the labels were aligned from left-to-right or right-to-left, top-to-bottom or bottom-to-top, and so on). This direction was judged from the negative label (‘least,’ ‘past,’ ‘worst’) to the positive label (‘most,’ ‘future,’ ‘best’).

**FIGURE 1:**
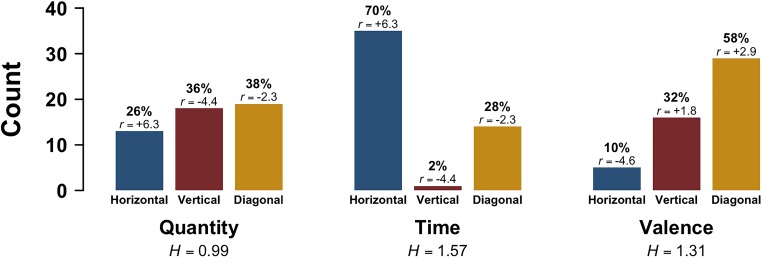
Experiment 1: Dominant Orientation (horizontal, vertical, diagonal) as a function of task; *r* indicates adjusted standardized Pearson residuals, which are based on the pooled data (participants contribute multiple data points), and *H* indicates Shannon entropy scores.

Figure 1 shows the distribution of Dominant Orientations across all three tasks. For the quantity task, 13 participants oriented the labels horizontally (26%), 18 vertically (36%), and 19 diagonally (38%). Responses to the time task were less variegated, with 35 participants orienting the two labels horizontally (70%); in contrast, only 1 participant (2%) oriented the labels vertically, and 14 participants oriented them diagonally (28%). In the emotional valence task, just 5 participants oriented the labels horizontally (10%), whereas 16 participants oriented them vertically (32%), and 29 diagonally (58%). A simple Chi-squared test of independence^1^ suggests that the data were incompatible with the null hypothesis that Dominant Orientation was independent from task [*χ^2^*(4) = 47.8, *p* < 0.0001, Cramér’s *V* = 0.40].

Adjusted standardized Pearson residuals from the Chi-squared test can be used to assess which particular cells are reliably over- or under-represented. We only discuss residuals with an absolute value larger than |2| (a commonly used cut-off; see Levshina, 2015: 220–221). These residuals show over-representation of vertical responses for the quantity task (+2.6), and over-representation of horizontal responses for the time task (+6.3). For the time task, vertical (-4.4) and diagonal responses (-2.3) were significantly under-represented. For the emotional valence task, diagonal responses were over-represented (+2.9), and horizontal responses under-represented (-4.6).

In an additional analysis, we grouped together horizontal and vertical (‘single-axis’) responses across the three tasks and compared these with diagonal responses, which use two axes simultaneously. There were slightly more single-axis responses (*N* = 88, 59%) than diagonal responses in the dataset (*N* = 62, 41%) (binomial test of equal proportions, *p* = 0.04).

Shannon entropy^2^ can be used to quantify the degree to which participants’ responses were overall more or less variegated for each of the tasks (a higher entropy indicates a greater diversity in responses). This measure shows that the quantity (*H* = 1.57) and emotional valence tasks (*H* = 1.31) showed the most variegated response pattern. The time task had the least variegated response pattern (*H* = 0.99).

What about the Dominant Direction within each Dominant Orientation? For horizontal responses, participants overall showed a strong preference for left-to-right responses: pooled across the three different experimental tasks, there were 56 left-to-right responses (90%), and only 6 right-to-left responses (10%). If we use Shannon entropy to calculate the diversity in responses (left-to-right versus right-to-left) per task, the quantity task had the highest entropy (*H* = 0.89), followed by emotional valence (*H* = 0.44), and then time (*H* = 0.18). This suggests that the horizontal direction is relatively less specified for quantity and emotional valence than it is for time. For vertical responses, the predominant order was down-to-up, with 84 responses having this direction across tasks (87%), compared to only 13 up-to-down responses (13%). A look at entropies shows that responses were most vertically consistent for quantity (least diversity, *H* = 0.18), followed by emotional valence (*H* = 0.68) and then time (*H* = 0.84).

Finally, for diagonal responses, there was more of a preference for a left-to-right direction in the quantity task (89%, *N* = 17) compared to the time (79%, *N* = 11) and emotional valence tasks (72%, *N* = 21). Diagonal responses were furthermore more likely to be down-to-up oriented in the quantity task (89%, *N* = 17), followed by the time (71%, *N* = 10) and emotional valence tasks (52%, *N* = 15).

#### Qualitative Results (Interview)

We recorded and transcribed all responses to the post-experiment interview. The resultant transcripts were then coded for several characteristics (see OSF repository for full description and codebook). Because this section is exploratory, we do not report the results of any confirmatory hypothesis tests.

When asked about their response to the quantity task, 21 out of 50 participants (42%) mentioned concepts related to mathematics (e.g., “normal *x* and *y* graph,” “grid with coordinates”). Interestingly, these participants were more likely to have responded diagonally in the quantity task (52%, 11 out of 21) than the 29 participants who did not mention mathematical concepts (28%, 8 out of 29). Only 3 participants (6%) mentioned cultural reading and writing conventions. In addition, 6 participants (12%) referenced embodied, environmental correlations where an increase in quantity is correlated with an increase in verticality, such as “a container” or “something that fills up from the bottom.” Of these participants, 4 responded vertically to the quantity task (4 out of 6, 67%), more than the proportion of participants who did not reference environmental correlations (14 out of 44, 32%).

In interview responses to the time task, 19 out of 50 participants mentioned timelines (38%). Of these participants, all but one responded horizontally (*N* = 18, 95%). In contrast, participants who did not mention timelines were somewhat less likely to respond horizontally (17 out of 31, 55%). Some of these participants also responded diagonally (13 out of 31, 42%), and one participant responded vertically (3%). Furthermore, 7 participants referred to cultural reading and writing conventions (14%), and 4 participants (8%) referenced numbers or mathematical concepts (e.g., “negative numbers,” “graph”).

For the emotional valence question, not a single person referenced timelines. In contrast, 6 participants referenced cultural reading and writing conventions (12%), and a total of 18 participants referenced mathematical concepts (36%). Of these 18 participants, 13 (72%) responded diagonally, 2 (11%) responded vertically, and 3 (17%) responded horizontally. On the other hand, participants who did not mention mathematical concepts were less likely to respond diagonally (50%, *N* = 16) and more likely to respond vertically (44%, *N* = 14) (horizontal: 6%, *N* = 2).

### Discussion

The quantitative results from Experiment 1 reveal a dominant down-to-up vertical representation for quantity, in agreement with some studies (Tversky et al., 1991; Winter and Matlock, 2013) but not others (Fischer and Campens, 2009; Holmes and Lourenco, 2012). This result might be explained by the proposal that vague quantities expressed linguistically are conceptualized vertically, whereas exact numerals are conceptualized horizontally (Winter et al., 2015b). We return to this proposal in Experiment 3. Furthermore, a number of participants in the quantity interview referenced embodied, environmental correlations (e.g., “something that fills up from the bottom”), a majority of which also responded vertically.

For time, we found a left-to-right horizontal representation to be dominant, in line with previous research (Tversky et al., 1991; Boroditsky, 2001; Fuhrman et al., 2011; Leone et al., 2018). The qualitative data suggest that many participants imagined timelines when responding to this task, and those participants who explicitly mentioned timelines were also more likely to respond horizontally.

Moving on to emotional valence, the most common response type was diagonal with a left-to-right directionality, an unexpected result based on the literature, which has more often found the vertical axis to be dominant (e.g., Crawford et al., 2006; Brunyé et al., 2012; Damjanovic and Santiago, 2016). A look at the direction of responses along the vertical axis paints a complex picture: diagonally oriented responses had both down-to-up and up-to-down directions, subverting our expectation that down-to-up responses would predominate (e.g., Meier and Robinson, 2004; Casasanto and Dijkstra, 2010; Seno et al., 2013). This chimes with the lack of systematicity reported for diagonal responses by Tversky et al. (1991). Overall, participants were most consistent in their association between time and the horizontal axis.

For Experiment 2, we intended to replicate the results of Experiment 1 with improved methodology. First, the fact that the response box in Experiment 1 contained axes may have primed participants to think of mathematical graphs, as suggested by the fact that many participants referenced mathematical concepts in their interviews. For this reason, we discarded the box, as well as the axes displayed inside each box. Instead, Experiment 2 used a blank paper, similar to Tversky et al. (1991). Moreover, we made the orientation of the response paper truly vertical by using a vertical stand. The reason for this change was that, as Winter et al. (2015b) note, many SNARC-like tasks purporting to find ‘vertical’ SNARC effects (e.g., Gevers et al., 2006; Müller and Schwarz, 2007; Shaki and Fischer, 2012) actually use sagittal response setups, where the ‘up’ button is further away from the ‘down’ button along the transversal plane in relation to the participant. We also used A3 rather than A4 paper, enlarging the response space.

Furthermore, we changed the words in the time task so they mirrored those used in the quantity and emotional valence tasks, as it was apparent that ‘past’ and ‘future’ do not possess the same kind of ‘oppositeness’ as the superlatives ‘least,’ ‘most,’ ‘worst’ and ‘best’^3^. Furthermore, we increased the number of words participants marked in each task from two to four. For time, these additional words were ‘earlier’ and ‘later,’ for quantity they were ‘less’ and ‘more,’ and for emotional valence they were ‘worse’ and ‘better’. This change was made because, with only two words per task in Experiment 1, it was not clear whether participants’ responses truly reflected axial conceptualisations. Using four words gave participants more freedom to structure their responses non-axially (i.e., not in a straight line), or in a non-linear order (e.g., in an order other than ‘worst,’ ‘worse,’ ‘better,’ ‘best’). Finally, the study’s instructions were read out verbally rather than written down, so the spatial position of these instructions could not bias responses.

## Experiment 2

### Participants

Sixty two native English-speaking adults (34 female, 28 male; 52 right-handed, 10 left-handed) volunteered to participate in the study. None had participated in Experiment 1. Data from one participant was discarded on the basis that they guessed the aims of the experiment correctly, leaving 61 participants (33 female, 28 male; 51 right-handed, 10 left-handed).

### Procedure

A3 paper was affixed to one transparent Deflecto 48011 A3 landscape stand-up sign holder (42.1 × 12.1 × 29.8 cm) with white Blu Tack. All participants completed three tasks sitting down with the stand positioned in front of them on a table. Each task involved marking four words onto this paper, with a new piece of paper being used for each task. The quantity task involved the words ‘least,’ ‘less,’ ‘more,’ and ‘most,’ the time task involved the words ‘earliest,’ ‘earlier,’ ‘later’ and ‘latest,’ and the emotional valence task involved the words ‘worst,’ ‘worse,’ ‘better,’ and ‘best’. The order in which these tasks were completed was randomized, which was thought to be another improvement upon Experiment 1, where the order of the tasks was fixed. The order of words within each task was also randomized.

Before beginning the study, participants were verbally read a list of instructions. Prior to responding to each task, participants were verbally informed of the four words they would be marking. These words were repeated once, and participants were told they could ask to hear them again as many times as they liked. Participants were instructed to mark the exact position of each word anywhere on the paper with an X, writing out the word in full next to each X. Marking each word with an X allowed us to perform continuous analyses of the positions in which participants chose to mark each word, which was not possible in Experiment 1, where participants instead marked the initial of each word (e.g., ‘L for LEAST’). The post-experiment interview procedure was identical to Experiment 1.

### Results

#### Quantitative Results (Placement Task)

Now that there were four words per task, we needed first to establish whether participants used a consistent axial orientation to begin with. Out of the 183 task responses collected overall, 99 (54%) were oriented along an axis (84 were not, 46%). This figure was roughly the same across all three tasks (quantity: 55%; time: 58%; emotional valence: 51%), and a simple Chi-squared test indicates no reliable difference of axis consistency across tasks [*χ^2^*(2) = 0.53, *p* = 0.77, Cramér’s *V* = 0.05]. In the following categorical analyses, we only use those responses that had a determinable axis orientation (horizontal, vertical, diagonal).

**FIGURE 2:**
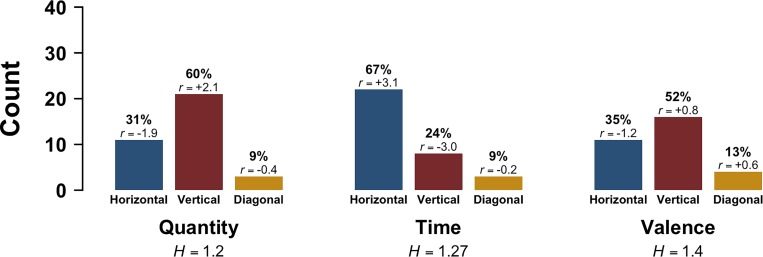
Experiment 2: Dominant Orientation (horizontal, vertical, diagonal) as a function of Task; *r* indicates adjusted standardized Pearson residuals, which are based on the pooled data (participants contribute multiple data points), and *H* indicates Shannon entropy scores.

For Dominant Orientation, we consider only the subset of responses that used a consistent direction (e.g., responses which marked the words in a linear order from ‘least’ to ‘less’ to ‘more’ to ‘most’). Figure 2 shows the distribution of Dominant Orientations across all three tasks. Participants were most likely to orient their responses to the quantity task along the vertical axis (60%), followed by the horizontal (31%) and then the diagonal (9%). For time, participants were most likely to respond along the horizontal axis (67%), followed by the vertical (24%) and then the diagonal (9%). Finally, for emotional valence, there also was a preference for the vertical axis (52%), with only 35% of responses being oriented horizontally and 13% diagonally. A Chi-squared test shows that the Dominant Orientation of the response differed reliably across tasks [*χ^2^*(4) = 11.07, *p* = 0.026, Cramér’s *V* = 0.24).

A look at the standardized residuals of this Chi-squared test shows that the vertical axis was over-represented for the quantity task (+2.1), and that the horizontal axis was relatively under-represented (-1.9). For the time task, the horizontal axis was over-represented (+3.1) and the vertical axis under-represented (-2.9). The pattern for emotional valence was similar to quantity, although less pronounced and overall more variegated (no standardized residual > |2|).

Across the board, responses were more variegated than in Experiment 1, as indicated by overall higher entropy values. Entropy was lowest for time (*H* = 1.2), followed by quantity (*H* = 1.27) and emotional valence (*H* = 1.4).

Comparing single-axis (horizontal and vertical) versus diagonal responses showed that there were also many more single-axis responses: 89 responses (90%) were single-axis compared to only 10 diagonals (10%) (binomial test *p* < 0.001).

Within the horizontally oriented responses, participants responded predominantly left-to-right (*N* = 48, 92%), compared to right-to-left (*N* = 4, 8%). Entropy values show that for the horizontal axis, there was less variation for time (*H* = 0.41) than for quantity (*H* = 0.59). The horizontal entropy for emotional valence was 0 since all horizontal responses were oriented left-to-right. For the vertically oriented responses, the pattern was predominantly down-to-up (*N* = 48, 86%), compared to up-to-down (*N* = 8, 14%). For the vertical axis, quantity responses were the least variable (*H* = 0.25), followed by emotional valence (*H* = 0.44) and time (*H* = 1.0).

**FIGURE 3:**
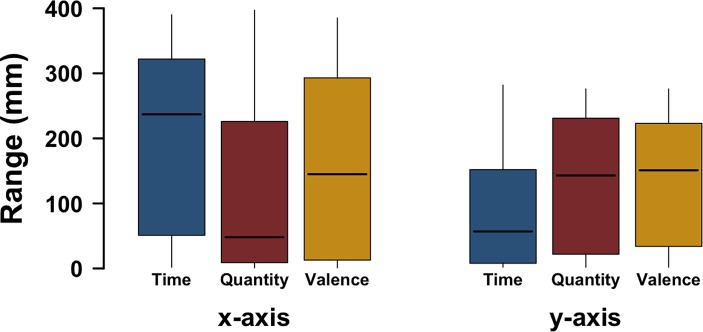
Experiment 2: Range of responses (smallest to largest) along the *x*-axis and the *y*-axis for each of the tasks; whiskers represent the largest/smallest number within 1.5 times the interquartile range extending from the box.

Since each participant marked labels on a continuous scale (millimeters), Experiment 2 also affords being analyzed using an approach that does not rely on manual annotation. We used the range as a statistical measure of spread, computing the *x*-axis range (from the leftmost to the rightmost data point) and *y*-axis range (from the lowest to the highest data point) separately for each trial. We analyzed these range values with a linear mixed effects model with axis (*x* versus *y*) and task (quantity, time, emotional valence) as fixed effects, including their interaction, and with random intercepts for subjects, as well as a random slope for by-participant variation in axis use. Likelihood ratio tests revealed a reliable interaction between axis and task [χ^2^(2) = 33.40, *p* < 0.0001]. This shows that which axis has larger ranges differs depending on task. The model fits also show that responses to the time task were much more horizontally (fitted value: 200 mm) than vertically extended (88 mm). In the quantity task, responses were slightly more vertically (132 mm) than horizontally extended (124 mm). Finally, in the emotional valence task, responses were slightly more horizontally extended (162 mm) than vertically extended (133 mm). Figure 3 shows boxplots for the ranges.

#### Qualitative Results (Interview)

A qualitative analysis of interview responses showed that for the quantity question, 4 participants referenced timelines (7%) and 4 referenced cultural reading and writing conventions (7%). A total of 10 participants (16%) mentioned environmental correlations in some fashion. These 10 participants were also much more likely to have responded vertically in the quantity task (8 out of 10, 80%), whereas only 25% of the participants who did not mention environmental correlations responded vertically (13 out of 51). A total of 6 participants (10%) mentioned mathematical and number-related concepts.

For the time question, 21 out of 61 participants (34%) referenced timelines. These participants were also much more likely to have responded horizontally in the time task: 16 of the 21 participants (76%) who mentioned timelines in their interview responded horizontally, compared to just 6 of the participants who did not mention timelines (15%; out of 40). Only 2 participants (3%) mentioned cultural reading and writing conventions, and just 2 participants mentioned mathematical or number-related concepts (3%).

In response to the emotional valence question, 3 participants referenced timelines (5%), 7 participants referenced mathematical or numerical concepts (11%), and 3 participants referenced cultural reading and writing conventions (5%).

### Discussion

Overall, participants were slightly more likely to use a consistent axial orientation in their responses than not, but many participants chose not to. Across all three tasks, participants tended to respond with single axes (horizontal, vertical) rather than combining two axes in diagonal responses.

The results for the quantity task confirm those found in Experiment 1; namely, we found a down-to-up vertical representation to be dominant. In another parallel with Experiment 1, interview responses to the quantity task contained references to environmental correlations, and a majority of these participants also responded vertically. Also in line with Experiment 1, time was primarily represented horizontally from left to right, and interview responses reveal that timelines consciously motivated many of these horizontal responses. Again, responses to the time task were the most consistent across the three domains.

There were far fewer diagonal responses compared to Experiment 1, which may have resulted from using blank paper rather than a box containing axes. These axes may have primed participants to think of graphs with both an *x*-axis and a *y*-axis. In support of this interpretation, fewer participants in Experiment 2 referenced mathematical concepts in relation to both emotional valence and quantity tasks.

We now move on to Experiment 3. As has been noted, some studies have found vertical effects for quantity, others horizontal effects. In Experiment 3 we investigate whether the prevalence of a particular representation differs depending on how quantity is represented. So far, Experiments 1 and 2 have shown that participants were more likely to select the vertical axis to represent quantity words. In Experiment 3 we investigate exact numerals.

## Experiment 3

### Participants

Forty one native English-speaking adults (22 male, 19 female; 32 right-handed, 9 left-handed) volunteered to participate in the study. 20 had participated in Experiment 1 prior to this, completing Experiment 3 directly afterward. The remaining 21 participants completed both Experiment 2 and Experiment 3 in one session, with tasks from both experiments randomized together.

### Procedure

The procedure here was identical to Experiment 2, except participants were instructed to place the numbers 2, 4, 7, and 9. Participants were not told what these numbers were supposed to ‘mean’ (e.g., whether they represented quantities, list items and so on). Because 1 and 10 are endpoints of a prototypical 1–10 number range, and because 5 is the midpoint of this range, these numbers were avoided so as not to prime a linear response. The intervals between each number were also deliberately unequal (as opposed to, e.g., 2, 4, 6, 8) for this same reason.

### Results

#### Quantitative Results (Placement Task)

**FIGURE 4:**
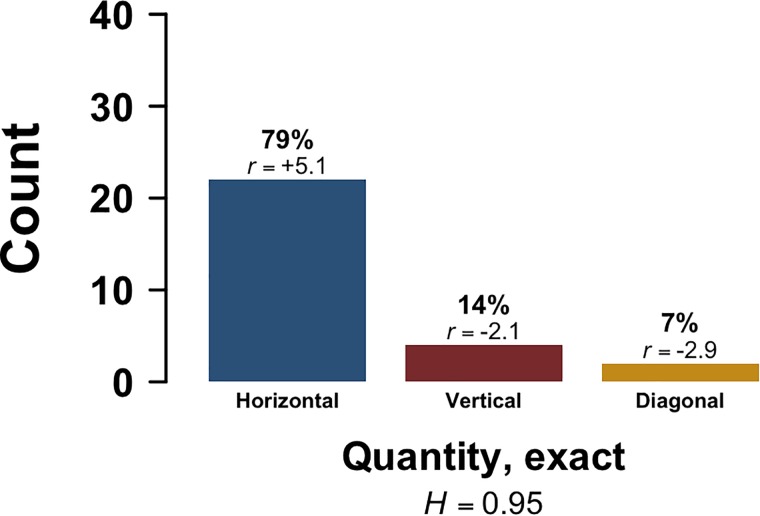
Experiment 3: Dominant Orientation (horizontal, vertical, diagonal) for the exact quantity task (placing the numerals 2, 4, 7, and 9); *r* indicates adjusted standardized Pearson residuals, and *H* indicates Shannon entropy scores, both of which are based on only the data from this task.

In Experiment 3, 28 out of 41 (68%) participants placed the numbers in a way that displayed a consistent axial orientation. Of these 28 responses, 22 were horizontal (79%), 4 were vertical (14%), and 2 were diagonal (7%). A Chi-squared test reveals that these observed counts were relatively unexpected under the null hypothesis of equal proportions [*χ^2^*(2) = 27.5, *p* < 0.0001, Cramér’s *V* = 0.68], with adjusted standardized Pearson residuals indicating horizontal responses to be over-represented (+5.1). In contrast, vertical (-2.1) and diagonal responses (-2.9) were under-represented. Shannon entropy shows that the diversity of responses for the number task in Experiment 3 (*H* = 0.95) was lower than for the quantity word tasks in Experiment 1 (*H* = 1.57) and Experiment 2 (*H* = 1.29). Figure 4 shows the distribution of counts observed in Experiment 3.

If we pool data from Experiment 3 and Experiment 1, a Chi-squared test shows that Dominant Orientation differed by experiment (quantity words E1 versus exact numerals E3) [*χ^2^*(2) = 20.4, *p* < 0.0001]. A similar result is obtained when comparing Experiment 3 with Experiment 2 [*χ^2^*(2) = 14.8, *p* = 0.0006].

Adjusted standardized Pearson residuals show that in the comparison of Experiment 3 and Experiment 1, horizontal responses were over-represented in the exact numeral task (+4.5) compared to the quantity word task. In addition, diagonal responses were over-represented in the quantity word task (+2.9) compared to the exact numeral task, and the same goes for vertical responses, although not as strongly (+2.0). The same comparison between Experiment 3 and Experiment 2 shows again that horizontal responses were over-represented in the exact numeral task (+3.7) compared to the quantity word task. Moreover, vertical responses were significantly more likely in the quantity word task (+3.7) than in the exact numeral task.

#### Qualitative Results (Interview)

Analysis of the interview data showed that 8 out of the 41 participants (20%) mentioned timelines. Of these 8 participants, 7 (88%) responded horizontally, compared to just 45% (15 of 33) participants who did not reference timelines. Additionally, in contrast to the quantity word task in Experiments 1 and 2, some participants mentioned number lines (*N* = 7, 17%). 3 participants also mentioned cultural reading and writing conventions (7%), and a total of 4 participants (10%) mentioned mathematics-related concepts.

### Discussion

Participants in Experiment 3 primarily chose to represent numerals horizontally from left to right. This stands in contrast to our results for vague quantity words such as ‘more’ and ‘less,’ which in Experiments 1 and 2 were represented primarily from down to up along the vertical axis. Thus, for quantity there is no one spatial conceptualization that trumps all others, but the precise conceptualization depends on the *kind* of quantity invoked.

Some participants referenced timelines in response to the exact numbers task, and these participants were more likely to respond horizontally compared to those who did not reference timelines. This could in part be due to carry-over effects from Experiments 1 and 2: all respondents who also participated in Experiment 1 completed the time task before the exact numbers task, and the randomization of task order in Experiment 2 meant that some of these participants completed the time task before the numbers task. Nevertheless, there is reason to believe that timelines could contribute to a horizontal representation of numbers, as the numbers on timelines (e.g., representing dates) do normally increase from left to right.

We have seen that, overall, participants in Experiments 2 and 3 preferred to structure their responses using single axes (horizontal, vertical), rather than using a diagonal or non-axial representation. However, it is possible that this choice was primed by the rectangular shape of the response paper, whose sides resemble *x*- and *y*-axes. The rectangular shape of the paper may have also primed participants to think of axis-based representations such as timelines and mathematical graphs, which may have influenced how they responded to each task. For these reasons, we conducted a fourth experiment where participants placed words inside a circle rather than a rectangle. In addition, we gathered data via a computer to remove connotations of reading and writing that may come with pen-and-paper tasks such as those used in Experiments 1–3.

## Experiment 4

### Participants

One hundred and twenty two native English-speakers (76 male, 46 female; 110 right-handed, 11 left-handed, 1 other) volunteered to participate in the study. None had participated in Experiments 1–3.

### Procedure

Qualtrics (2018) was used to administer the web experiment, which was distributed via Amazon Mechanical Turk. The main portion of the experiment consisted of four tasks created using the ‘Heat Map’ question type of the Qualtrics survey design software. Participants had to mark four words or numbers per task inside a circle displayed on the screen. Participants did this by clicking or touching the screen (depending on what kind of device they were using) in the order that the words/numbers were presented. We re-used the stimulus words from Experiments 2 and 3. The order of the four tasks and the order of the words within each task were randomized. Following this, participants were asked if they made any mistakes when responding to the previous four tasks. Any responses that were claimed to be mistakes were removed from further analyses.

The rest of the study was identical to Experiments 1–3 with one exception: participants were asked to state other languages that they could speak apart from English. This information was used to exclude participants who were familiar with languages with different reading and writing conventions to English (e.g., Hebrew, Mandarin), which could have influenced their responses.

### Results

#### Quantitative Results (Placement Task)

Overall, 166 (45%) of responses were oriented along a consistent axis, compared to 201 (55%) that were not. The choice of whether or not to use an axis varied somewhat across tasks (quantity: 51%, exact numerals: 35%, time: 43%, emotional valence: 51%). However, a Chi-squared test revealed no reliable difference across tasks [*χ^2^*(3) = 6.23, *p* = 0.1, Cramér’s *V* = 0.13].

**FIGURE 5:**
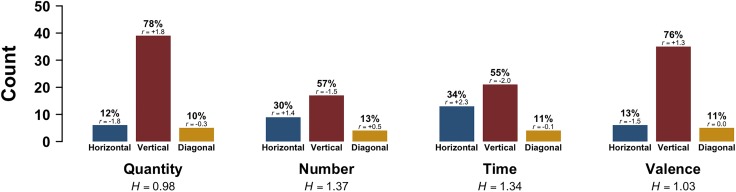
Experiment 4: Dominant Orientation (horizontal, vertical, diagonal) as a function of Task; *r* indicates adjusted standardized Pearson residuals, which are based on the pooled data (participants contribute multiple data points), and *H* indicates Shannon entropy scores.

Figure 5 shows the distribution of Dominant Orientations across the four tasks. Most responses in the quantity task were vertical (78%), followed by horizontal (12%) and diagonal responses (10%). Responses to the exact numerals task were also more likely to be vertical (57%), followed by horizontal (30%) and then diagonal (13%). Similarly, responses to the time task were more likely to be vertical (55%), followed by horizontal (34%) and then diagonal (11%). Finally, for the emotional valence task, participants were also more likely to respond vertically (76%), followed by horizontally (13%) and diagonally (11%). This time, however, a Chi-squared test failed to show a reliable difference of Dominant Orientation across tasks [*χ*^2^(6) = 10.56, *p* = 0.1, Cramér’s *V* = 0.18].

In terms of axial responses, responses were least variegated for the quantity task (*H* = 0.98), followed by the emotional valence task (*H* = 1.03). In contrast, responses to the exact numerals task (*H* = 1.34) and the time task (*H* = 1.34) were considerably more variegated. In addition, a comparison of single-axis (horizontal and vertical) versus diagonal responses shows that there were far more single-axis responses (*N* = 146, 18%) than diagonal responses (*N* = 18, 11%) (binomial test: *p* < 0.001).

For horizontally oriented responses, most responses had a left-to-right direction (*N* = 36, 86%), with only a small number having a right-to-left direction (*N* = 6, 14%). For vertical responses, most responses were structured with a down-to-up direction (*N* = 59, 73%), with only 27% (*N* = 22) being up-to-down. A look at the entropy values shows that the direction of horizontal responses was more variegated for quantity (*H* = 0.81) and emotional valence (*H* = 0.76) than for exact numerals (*H* = 0.44) and time (*H* = 0.37). We see the reverse pattern when we look at the entropy values for vertical responses: here, quantity was the least variegated (*H* = 0.59), followed by emotional valence (*H* = 0.74), whereas exact numerals (*H* = 0.92) and time (*H* = 0.99) were much more variegated.

There were also many responses in this experiment that appeared to have a circular structure. Overall, there were 114 circular responses (41%), compared to 166 responses that used an axis (59%) (binomial test, *p* = 0.59). One issue here is that because participants marked only four words, we cannot say with confidence that they structured their response circularly. To investigate this issue, we can look at whether participants structured their allegedly ‘circular’ responses consistently clockwise or counter-clockwise. The results from this analysis show that 48% (*N* = 55) of circular responses were structured in a consistent order, compared to 52 (*N* = 59) of responses that were not (binomial test: *p* = 0.78). This suggests that many responses initially coded as ‘circular’ may not have been circular for the participant (e.g., they may have been random). Of the circular responses that were structured in a consistent order, 69% (*N* = 38) were structured in a clockwise direction, and 31% (*N* = 17) were structured counter-clockwise.

**FIGURE 6:**
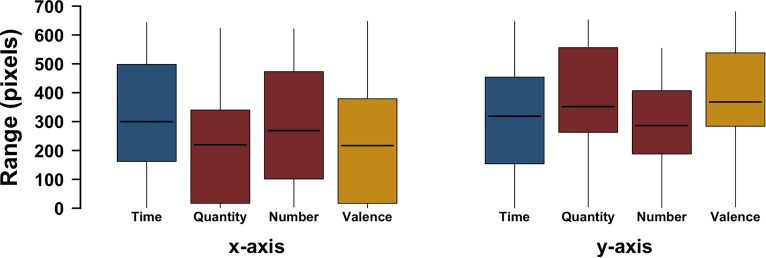
Range of responses (smallest to largest) along the *x*-axis and the *y*-axis for each of the tasks; whiskers represent the largest/smallest number within 1.5 times the interquartile range extending from the box.

Similar to Experiment 2, we also analyzed the range (minimum and maximum) across axes and tasks with a linear mixed effects model (fixed effects: axis, task, axis ^∗^ task interaction; random effects: random subject intercepts, random by-subject axis slopes). As in Experiment 2, likelihood ratio tests revealed a reliable interaction between axis and task [*χ*^2^(3) = 51.09, *p* < 0.0001]. This shows that which axis has larger ranges differs depending on task. An analysis of the model’s predictions reveals that this interaction largely stems from the quantity task being more extended vertically (fitted value: 376px) than horizontally (209px), which was also the case for emotional valence (predicted vertical range: 367px; horizontal range: 226px), but not for time (horizontal: 319px; vertical: 307px) and number (horizontal: 287px; vertical: 296px), for which the difference between the axes was very small. Figure 6 shows a boxplot of the range per task.

#### Qualitative Results (Interview)

For the quantity task, only 3 out of the 122 participants (2%) mentioned numbers and mathematics-related concepts. In addition, 7 participants (6%) mentioned environmental correlations, and no participants mentioned timelines or cultural reading and writing conventions. A more common interview response in this task was to talk about starting from the center of the circle, extending outward to the outer edges (*N* = 14, 11%), which we will call ‘radial’ responses. For instance, one participant said that they thought “most seemed more appropriate in the middle of the circle, where as [sic] close to the edge seemed to resemble less”. A similar response was to mention that the center of the circle reminded them of a bullseye or target (*N* = 1, 1%). Finally, 4 (3%) participants mentioned clocks in response to the quantity task.

For the exact numerals task, the most common response was to reference clocks (*N* = 19, 16%); for example, one participant said that they structured their response “based on the numbers of a clock face.” Furthermore, 5 (4%) participants referenced mathematical concepts other than numbers, 1 (1%) participant mentioned environmental correlations, and no participants mentioned timelines or cultural reading and writing conventions. Moreover, 6 (5%) participants talked in radial terms, with 3 (2%) participants making reference to targets or bullseyes.

In their interview responses to the time task, only 6 (5%) participants mentioned timelines, whereas 9 (7%) participants mentioned clocks. 2 (2%) participants referenced numbers and mathematical concepts, 1 (1%) participant referenced environmental correlations, and 1 (1%) participant referenced cultural reading and writing conventions. Slightly more participants (*N* = 6, 5%) talked in radial terms. No participants made reference to targets or bullseyes.

For emotional valence, the most common interview response was to say that they structured their task response radially (*N* = 14, 11%), with 3 (2%) participants referencing bullseyes and targets. 3 (2%) participants talked about timelines, 1 (1%) participant mentioned environmental correlations, and no one mentioned timelines or cultural reading and writing conventions. Finally, 4 (3%) participants said that their task response was influenced by clocks.

Where participants talked about clocks in their interview responses, we might expect them to also structure their task response in a circular, clockwise direction. This was the case: in all 33 instances where a participant referenced clocks, their response was also circular. Of the 33 instances that had a consistent direction, 90% (*N* = 19) were structured in a clockwise direction, whereas just 10% (*N* = 2) were structured in a counter-clockwise direction. In comparison, if a participant did not reference clocks, they were more likely to respond axially: 67% (*N* = 166) of these responses were axial, whereas 33% (*N* = 81) were circular.

### Discussion

Numerically, the categorical analysis showed a clear pattern with the vertical axis being over-represented for quantity and emotional valence responses. However, we failed to find a task effect using the same methods used for the other experiments (Chi-square test). We believe that this unreliable outcome stems partly from the fact that there were fewer axial responses overall, primarily due to the uptake in circular responses that seem to have been inspired by clock faces. Furthermore, we suspect that in the Chi-square test analysis, it is specifically the evenly distributed number of diagonal responses (which were also overall low in number) that obscure the overall result. When a Chi-square test is performed on vertical and horizontal responses only, there is a reliable difference across tasks [*χ*^2^(3) = 10.34, *p* = 0.02, Cramér’s *V* = 0.27], with horizontal responses being over-represented for the time task (standardized residual + 2.35). Because this analysis was not planned in advance, it is all the more important that the analysis of continuous pixels replicated the axis ^∗^ task interaction effect that was also obtained for Experiment 2. This continuous analysis showed that responses were more vertically extended for quantity and emotional valence than for exact numerals and time, similar to what was found in Experiment 2.

Previously strong horizontal orientations observed in the time (Experiments 1 and 2) and exact numerals tasks (Experiment 3) were much weaker in Experiment 4. We partly attribute this to the circular response area influencing participants to structure their responses in line with the layout of a clock face. This explanation is supported by the interview data, where many participants referenced clocks. In contrast, the vertical orientation observed for quantity in Experiments 1 and 2 remained intact in Experiment 4, which suggests that the association between quantity and vertical space may be less task-dependent. The durability of this vertical quantity association may be due to its purportedly embodied origins in environmental correlations (e.g., Lakoff, 1987; Fischer and Brugger, 2011; Winter et al., 2015a), which participants consistently referenced in their interview responses to the quantity task across Experiments 1, 2, and 4. In contrast, many participants stated that cultural representations such as timelines, clocks and number lines influenced their responses to the time and exact numerals task (see also Duffy, 2014). These cultural representations arguably have more in common with the visual placing of words required in our tasks, which could explain how the tasks themselves were able to shift participants’ responses.

## General Discussion

Abstract concepts tend to be grounded in space. However, not all conceptual domains are created equal: spatial representations differ across quantity, time, and emotional valence. Overall, our participants preferred the vertical axis for emotional valence and vague quantity words. Both of these mappings correspond to metaphorical expressions used by English speakers, as when talking about feeling ‘high’ or ‘low,’ or when describing ‘high’ and ‘low’ numbers. In our experiments, spatial representations of quantity also differed depending on what type of quantity is implied: whereas participants oriented vague quantity words vertically, they oriented exact numerals horizontally. Time, on the other hand, was mostly represented horizontally, except in Experiment 4, where the circular response area prompted a large number of participants to respond circularly, akin to a clock.

Interview responses for time suggest that timelines motivated many horizontal responses to this task. These qualitative results align with studies demonstrating the influence of cultural artifacts on spatial-temporal mappings (Tversky et al., 1991; Fuhrman and Boroditsky, 2010; Bergen and Chan Lau, 2012; Duffy, 2014). For the vague quantity words, several interview responses contained references to environmental factors, such as the correlation between quantity and height in the real world (e.g., containers filling up with liquid), and these participants were also more likely to have responded vertically. This lends some support to Lakoff and Johnson (1980, 1999) and Lakoff’s (1987) claims regarding the embodied, environmental origins of the vertical association between quantity and space (see also Fischer and Brugger, 2011; Winter et al., 2015a).

As predicted by Winter et al. (2015b), vague quantity words were more likely to be represented vertically than exact numerals. Because our study presented quantity words in isolation, we were able to show that vertical representations persist even when these quantities are not contextualized within a concrete situation (cf. Pecher and Boot, 2011). At present, there are two possible explanations as to why vague quantity words might be conceptualized vertically. For one, quantity words such as ‘more’ and ‘less’ are associated with language, in which people exclusively use vertical space to talk about quantities (e.g., ‘high number,’ ‘plummeting costs’). This is a metaphor-focused explanation. Alternatively, the verticality attached to vague quantity words may stem from the fact that, compared to exact numerals, these words express less precise quantities and tap into a more general sense of mass (see Holmes and Lourenco, 2012). This conception of mass may align with environmental correlations we see in the real world, such as water filling up a glass. More work is needed to unpick these two factors; we suggest contrasting numerals with exact numbers expressed in linguistic form (e.g., the number word ‘seven’).

In line with previous research, we found that time was oriented in a left-to-right manner (e.g., Tversky et al., 1991; Boroditsky, 2001; Fuhrman et al., 2011). This horizontal representation mirrors the dominant orientation of exact numerals in Experiment 3, just as timelines and number lines follow the same left-to-right trajectory. Thus, it is possible that time and number are closely related in cognition, at least when it comes to the horizontal axis. This is also suggested by the fact that in their interview responses, several participants referenced timelines when talking about number concepts. Furthermore, although it was not the prime concern of this study, we should note that we observed horizontal effects for both deictic time (‘past,’ ‘future’) and sequence time words (‘earliest,’ ‘earlier,’ ‘later,’ ‘latest’) (cf. Fuhrman et al., 2011; Kranjec and McDonough, 2011).

Our results for emotional valence were slightly less clear; responses to this task were overall more variable than for quantity or time. This could suggest that emotional valence is less established in spatial thought than quantity, and especially time. However, overall, responses to the emotional valence task tended to have a down-to-up orientation (as reported by, e.g., Tversky et al., 1991; Crawford et al., 2006; Brunyé et al., 2012), in line with metaphorical descriptions of good and bad emotions in English (e.g., ‘feeling down,’ ‘cheer up’). This aligns with Tversky’s (2011) prediction that the vertical dimension should be preferred for more evaluative concepts, whereas the horizontal dimension is more neutral: time is arguably a more neutral concept than emotional valence.

How do our results relate to Walker and Cooperrider’s (2016) continuity of metaphor hypothesis? These researchers found evidence in spontaneous gestures for the co-activation of different axes for time representations. Our results speak only indirectly to their conclusions, which were based on an analysis of naturally occurring gestures. However, we find that, at least when responding on a two-dimensional plane, participants prefer to stick to specific axes (horizontal, vertical) rather than orienting concepts diagonally. The fact that our computerized task (Experiment 4) showed overall fewer axial responses is not direct support for the simultaneous mental co-activation of axes, since most non-axial responses had either a circular structure, or no structure at all. Moreover, where there were a relatively large number of diagonal responses in Experiment 1, we found that these diagonal responses lacked systematicity (as reported by Tversky et al., 1991). Another problem with interpreting these diagonal responses is suggested by the fact that the sagittal axis is typically represented in two-dimensional graphs with a diagonal line extending from the bottom left to the upper right of the graph. This means that participants may have used diagonal lines to represent the sagittal axis, which is possible given that spatial representations of emotional valence (e.g., Solarz, 1960; Markman and Brendl, 2005) and quantity (see Winter et al., 2015b for a review) have also been reported along the sagittal axis. In general, it seems that in our tasks there was a strong pull to use single axes rather than a combination of axes.

To conclude, space is an immensely useful thinking tool which humans use to understand abstract concepts. Quantity, time and emotional valence can all be thought of as being situated along specific axes, but whether horizontal axes (time, exact numerals) or vertical axes (vague quantity words, emotional valence) are preferred depends on the particular domain of abstract thought. Whether one axis is dominant, and which axis is dominant, depends on the precise nature of the task, as well as the precise conceptual domain.

## Ethics Statement

This study was carried out in accordance with the recommendations of the British Association for Applied Linguistics (BAAL). The protocol was approved by the College of Arts and Law at the University of Birmingham. All subjects gave written informed consent in accordance with the Declaration of Helsinki.

## Author Contributions

GW and BW conceived the experiments. GW implemented the experiments. GW coded the data. BW performed the statistical analyses. GW and BW wrote the manuscript.

## Conflict of Interest Statement

The authors declare that the research was conducted in the absence of any commercial or financial relationships that could be construed as a potential conflict of interest.

## Footnotes

1 As each participant provides three data points (one for each task), the independence assumption of the Chi-squared test is violated. This violation, however, is not a strong one since it is impossible to have multiple data points from the same participants within a single cell or even within the same task. To show that our conclusions are not affected as a result of this, we created 1,000 random datasets where each participant only contributed one data point. The average Chi-squared value across these 1,000 random datasets was 18.03, much in excess of the critical Chi-squared value for *α* = 0.05 and df = 4 (for a 3 × 3 table), which is 9.5. It should furthermore be noted that a logistic mixed effects regression model with the dependent variable Dominant Orientation (horizontal versus vertical, excluding diagonal), a fixed effect for task (quantity, time, emotional valence) and a random intercept for subject also yields a significant effect of condition [likelihood ratio test: *χ^2^*(2) = 44.33, *p* < 0.0001]. For Experiment 2, a similar sanity check revealed more equivocal results. The randomization procedure showed the mean Chi-squared value (6.25) to be below the critical value of 9.5. However, a logistic mixed effects model with the same structure still yielded a reliable effect for horizontal versus vertical responses [*χ^2^*(2) = 24.27, *p* < 0.0001].

2 Shannon entropy is given by $H=-\sum_{i}^{R}p_{i}\times \log_{2}(p_{i})$ where *R* is the richness (the number of categories, in this case three) and p_i_ is the proportion of the *i*^th^ category.

3 One consequence of this was that the new terms, ‘earliest’ and ‘latest,’ were sequence time words, as opposed to ‘past’ and ‘future,’ which were deictic time words. So far, studies have found no consistent association between sequence time and the sagittal axis (Fuhrman et al., 2011; Kranjec and McDonough, 2011), whereas with deictic time they have (e.g., Kranjec and McDonough, 2011; Ulrich et al., 2012; Walker et al., 2017). However, to our knowledge, the literature does not suggest that this distinction matters when it comes to the horizontal and vertical axes.
